# ADVICE: A New Approach for Near-Real-Time Monitoring of Surface Displacements in Landslide Hazard Scenarios

**DOI:** 10.3390/s130708285

**Published:** 2013-06-27

**Authors:** Paolo Allasia, Andrea Manconi, Daniele Giordan, Marco Baldo, Giorgio Lollino

**Affiliations:** Geohazard Monitoring Group, CNR IRPI, Strada delle Cacce 73, Torino 10135, Italy; E-Mails: paolo.allasia@irpi.cnr.it (P.A.); daniele.giordan@irpi.cnr.it (D.G.); marco.baldo@irpi.cnr.it (M.B.); giorgio.lollino@irpi.cnr.it (G.L.)

**Keywords:** landslides, surface deformation, monitoring, early warning systems

## Abstract

We present a new method for near-real-time monitoring of surface displacements due to landslide phenomena, namely ADVanced dIsplaCement monitoring system for Early warning (**ADVICE**). The procedure includes: (i) data acquisition and transfer protocols; (ii) data collection, filtering, and validation; (iii) data analysis and restitution through a set of dedicated software; (iv) recognition of displacement/velocity threshold, early warning messages via SMS and/or emails; (v) automatic publication of the results on a dedicated webpage. We show how the system evolved and the results obtained by applying ADVICE over three years into a real early warning scenario relevant to a large earthflow located in southern Italy. ADVICE has speed-up and facilitated the understanding of the landslide phenomenon, the communication of the monitoring results to the partners, and consequently the decision-making process in a critical scenario. Our work might have potential applications not only for landslide monitoring but also in other contexts, as monitoring of other geohazards and of complex infrastructures, as open-pit mines, buildings, dams, *etc.*

## 1. Introduction

Landslide processes cause every year severe damages, as well as a large number of fatalities worldwide [1]. The identification and analysis of surface deformation plays an important role for understanding the evolution of landslide phenomena, and is vital in monitoring activities aimed at ensuring safety of people and/or infrastructures. Furthermore, the analysis of the surface evolution of an instable slope allows obtaining information that can be used for the correct design and the implementation of effective stabilization measures [2,3]. Nowadays, a wide spectrum of instruments is available to monitor topographic changes due to slope movements, allowing us to retrieve displacements with sub-centimetric accuracies at high temporal frequencies [4]. These monitoring systems are often based on a quantitative analyses of accurate measurements obtained by means of different sensors, including: Synthetic Aperture Radar Differential Interferometry (DInSAR [5–8]), Global Position System (GPS [9–12]), Robotized Total Stations and extensometer networks [13–16]. In some cases, Early Warning Systems (EWS) working in landslide scenarios are based on surface deformation measurements [17–19]. Warning and/or alarm thresholds are set on measured displacements and/or velocities, and critical values are defined by experts taking into account the landslide typology, the kind of monitoring instruments used, and the value exposed at risk.

**Figure 1.**
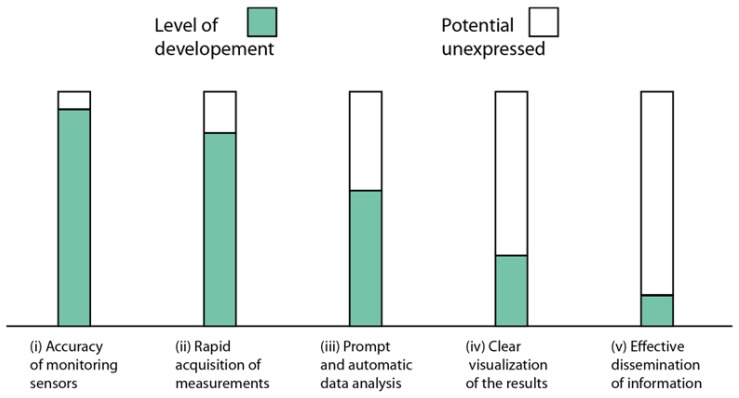
Bar-chart to schematically represent the main requirements of EWS in landslide scenarios. Green areas represent the current level of development achieved, white areas stay for unexpressed potential, respectively. In the context of EWS, the state-of-art of some elements already reached a high level of development (i and ii), while others have still a high potential (iii, iv, and v).

In civil protection contexts, the efficiency of EWS is a function of several terms, including: (i) accuracy of the instruments; (ii) rapid acquisition of measurements; (iii) prompt and automatic data analysis (including validation of the measurements); (iv) clear visualization of the results; (v) effective dissemination of the obtained information [20]. A large effort was accomplished in the last decades to develop and deploy accurate sensors and instruments, to set up automatic procedures, as well as to improve data transfer protocols and retrieve measurements from remote areas in real-time, or in times ranging from minutes to hours (hereinafter referred to as “near-real-time”). These technological developments allow us for an immediate detection of landslide activity, which is crucial to make timely decisions about safety in hazardous contexts [21]. On the other hand, the tools available for the exploitation, visualization, and subsequent divulgation of surface displacement measurements have still a high level of unexpressed potential, as their level of improvement (in this specific context) is far behind that reached by the available monitoring sensors. This generates a bottleneck in terms of efficiency between the availability of invaluable information based on surface displacement data and its use for early warning purposes in civil protection scenarios (see Figure 1).

In this work, we propose a new approach aiming to achieve an efficient procedure to monitor surface displacements in landslide scenarios. Following the experience gained in several monitoring contexts, including emergency scenarios [22–24], we developed a set of tools that passes automatically, and in near-real-time, from the acquisition of displacements data to the divulgation of the monitoring results via the Internet. This set of straightforward procedures is called ADVanced dIsplaCement monitoring system for Early warning (ADVICE), and includes: (i) data acquisition and transfer protocols; (ii) data collection, filtering, and validation; (iii) data analysis and restitution through a set of dedicated software; (iv) recognition of displacement/velocity threshold, early warning messages via SMS and/or emails; (v) automatic publication of the results on a dedicated webpage. In the following, we first show the operational principles of ADVICE. Secondly, we describe the application of our approach to a real emergency scenario, the Montaguto earthflow (southern Italy, *ca*. 100 km northeast from Naples). Finally, we discuss our results, mainly focusing on how the use of ADVICE has speed-up and facilitated the understanding of the landslide evolution, the communication of the monitoring results to the partners, and consequently the decision-making process in a critical landslide scenario.

## 2. ADVICE: Operational Principles

**Figure 2.**
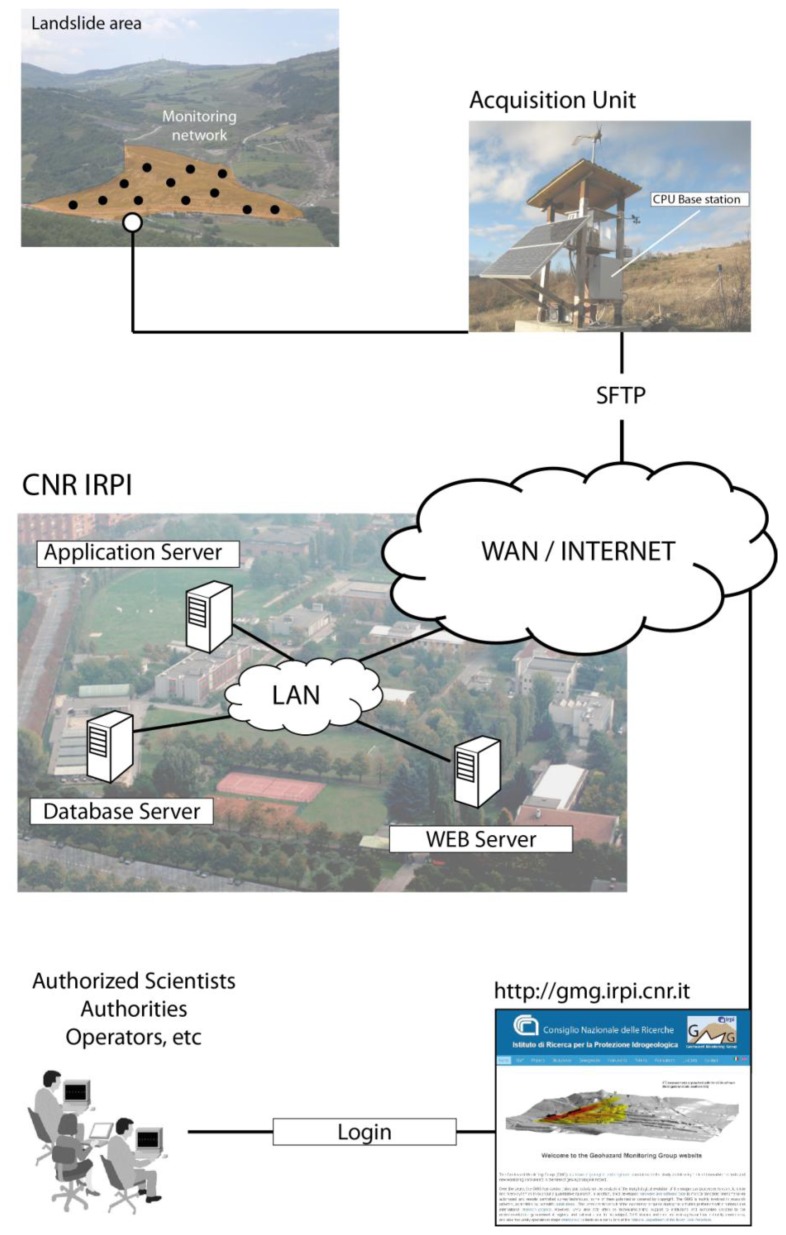
Rationale of the ADVICE system as applied for displacement monitoring via RTS in a landslide scenario.

**Figure 3.**
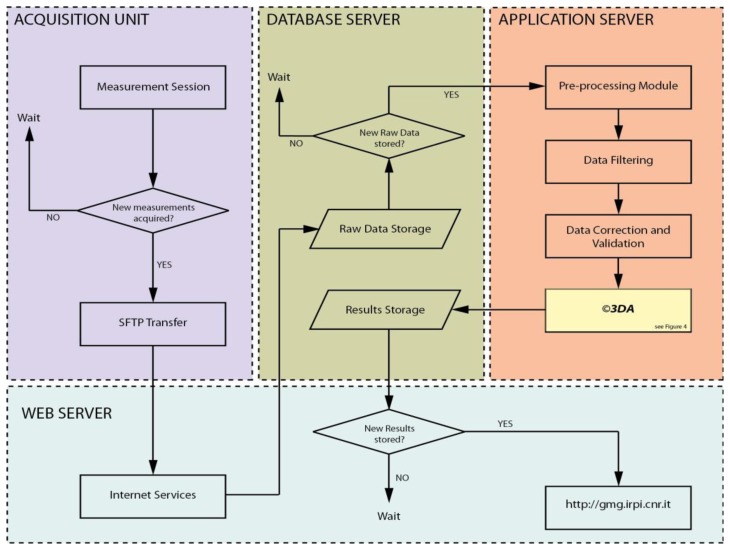
Detailed Flow Chart of the ADVICE key elements.

The rationale behind the near-real-time monitoring approach proposed in this work is outlined in Figure 2. The latter shows the key elements of ADVICE, while a more detailed flow-chart describing the procedure step-by-step is presented in Figure 3. The herein presented methods, applications, and results of ADVICE are referred to a monitoring network based on a Robotized Total Station (RTS) and a set of optical prisms (targets) installed in an instable slope area [25]. However the concept, as well as the key elements of the system proposed, can be applied also to different sensors and monitoring scenarios (see Section 4).

### 2.1. Acquisition Unit

The first stage is the data acquisition. In general, the basic features (acquisition parameters, time scheduling, power management, *etc.*) of different acquisition unit (e.g., RTS, extensimeter, or other monitoring instruments) are managed via ad-hoc proprietary software usually deployed in a base station installed directly at the monitored site. After the measurement sessions, data is then periodically downloaded and analyzed. However, when Internet connection is available (through GSM, GPRS, UMTS, dial-up or dedicated backbones), the local control station may be queried directly from a remote server, usually located at the monitoring division site, and transmitted automatically via secure file transfer protocol (SFTP).

### 2.2. Database and Application Server

After the file transfer procedure, data are uploaded and stored on a dedicated Database server, and are ready for the processing steps. The database structure (whose description is omitted for brevity), is built around three main entities: the observed phenomenon, the monitoring instrument, and the measurements. The Database server and the Application server are managed via a software (ADMIN) implemented in Visual Basic, which consists of several modules, including: (i) a data acquisition module, which manages the connection with the base station via the Internet (for raw data transfer); (ii) a database interface module, which reads the raw data and writes processed data into the database; (iii) a pre-processing module.

The data pre-processing module first converts raw data into the correct metric-scale and coordinates system. Taking into account the example of RTS, the instrument records two angular values (in radians) and a distance (in meters) in a local reference system, thus data need to be converted to the physical parameter of interest (displacement components, in meters) defined in a geographic coordinate system (e.g., WGS-84). Then, the database is filtered, by removing inconsistent data, noisy measurements, spikes, and handling missing values. After that, the data is corrected and validated by considering a set of predefined algorithms. For that regarding RTS data, one of the standard operations is to validate measurements by checking the position of reference targets (installed on stable areas), and by calculating scaling factors to compensate for optical distortions effects [25]. Validated data is passed to a set of specific software routines called ©3DA, which have been developed for an automated and advanced processing of the displacement measurements.

### 2.3. ©3DA—Three-Dimensional Displacement Analysis

**Figure 4.**
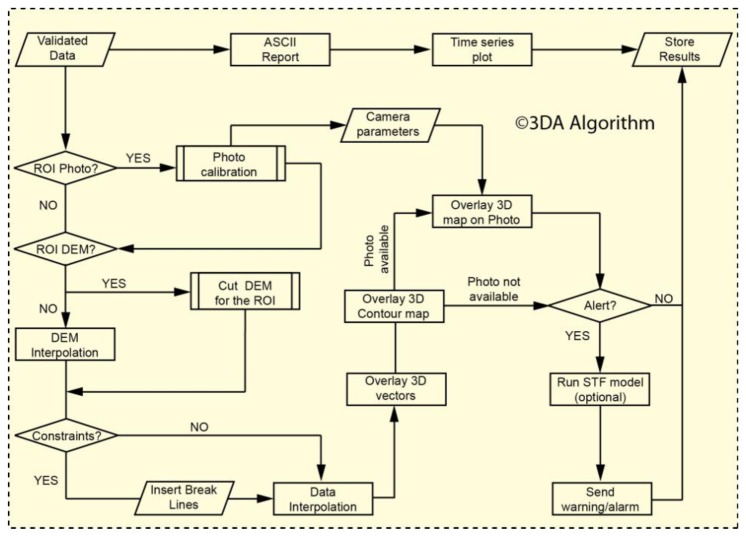
Detailed flow chart of the ©3DA Algorithm as implemented in Matlab^®^.

Surface displacement data are habitually presented either as a table containing numeric values relevant to a time interval, or in the form of bi-dimensional charts, where the measured quantity (baseline change, line-of-sight change, easting, northing, vertical motion, or some combination of the observed deformation components) is plotted *versus* time. By comparing the actual representations of displacement data to the results of pioneeristic monitoring activities in landslide scenarios, it is evident that differences are minimal [26,27]. The bi-dimensional charts might be rather straightforward to experienced people, who are used to deal with monitoring data; however, in real operative scenarios multidisciplinary teams work for civil protection purposes, and a rapid and accurate understanding of the deformation measurements as well as a clear and unambiguous representation of the data is vital to efficiently support decision makers. Thus, the main limitations of the bi-dimensional charts are: (i) a specific background-knowledge of the data and of the phenomena is usually required in order to read, understand and interpret the displacement results; (ii) the informative content of the data is usually distributed within a large number of outputs/documents; (iii) this representation is not suitable for a rapid and effective divulgation of the updated situation, especially to people not having basic knowledge of monitoring systems, but should act and/or react promptly (e.g., authorities, workers, population, *etc.*). For these reasons, we implemented a set of procedures to achieve Three-Dimensional Analysis in near-real-time (©3DA, [28]). ©3DA produces three-dimensional magnitude maps of a physical quantity (in this specific case, the deformation of the monitored targets in an arbitrary temporal interval) by interpolating the results of measurements over a given Region-Of-Interest (ROI). Moreover, at the measurement points ©3DA provides additional indications on the real intensity and direction of motion (*i.e.*, 3D vectors). The results of the ©3DA algorithm are projected on and referenced to a real (photo) and/or realistic (digital model) representation of the ROI. Figure 4 shows the flow-chart of the ©3DA algorithm, as implemented in Matlab^®^, a high-level programming language.

©3DA allows for a number of interpolation algorithms (e.g., nearest neighbor, bilinear, triangular, *etc.*) and has several built-in features that control the data import as well as the final graphical representation output. The selection of the spatial extension of the interpolation field as well as the interpolation grid step can be modified depending on the peculiarities of the monitored area. Moreover, information on the boundary and/or structural discontinuities (*i.e.*, break-lines) present in the monitored area can be included as input and then passed to the interpolation subroutine. The resulting deformation map consists in a representation of a 3D surface, where the contouring can be either relevant to the displacements with respect to the previous measurement cycle, the deformation rate, or the acceleration in the same time frame. Because the displacement contour maps are the result of an interpolation procedure, the map is most accurate near the target locations, *i.e.*, where the data is well constrained by the real measurements. However, depending on the phenomenological and/or logistical characteristics of the area of interest, if the monitoring network geometry and the point target distribution is appropriately planned, the interpolated intensity maps provide a good approximation of the spatial distribution of surface deformation. Moreover, by considering displacements in the three directions at a certain time, vector arrows representative of the real direction of motion in a given system of coordinates are associated to the monitored targets. The intensity of vector arrows is opportunely scaled to be clearly represented and overlain in the same output. We also implemented the possibility to overlay the 3D surface deformation maps on a digital model and/or photo of the monitored area. This solution can be very useful for end-users to immediately understand the current situation. The procedure for the overlaying of the vector on a photo is performed by knowing some basic photogrammetric parameters of the taken picture, as the coordinates of the camera (either local or geographic), tilting during the shot, viewing angle, *etc.* A specific subroutine with a Graphical User Interface was also implemented to adapt the visualization parameters and achieve the best overlay on the photo. The advantage of this representation is mainly that a new picture of the ROI can be taken from the same (or a new, more convenient) position, and then updated with higher frequencies with respect to a DEM of the area. This three-dimensional view is particularly suitable in cases of landslides that present rapid surface modifications. Furthermore, by using these outputs in a multitemporal analysis, it is possible to generate a movie to represent the landslide evolution over time (see repository). ©3DA will be further developed to integrate displacement measurements acquired via different monitoring instruments (GPS, extensometers, DInSAR data, inclinometers, *etc.*), in order to obtain an holistic representation of the deformation associated with a landslide event.

As mentioned in Section 2.1, the application server governs the whole ADVICE system through the ADMIN software. ©3DA subroutines have been compiled (by considering the Matlab^®^ Compiler facilities) and are treated by ADMIN as stand-alone executable (*i.e.*, a set of .exe files under a Windows operative system). This permits to have versatility from the measurement acquisition to the final production of displacement results, by permitting the integration of diverse software developed in different languages.

### 2.4. Web Server

In emergency situations, which can be relevant to the activation, re-activation, and/or acceleration of the landslide phenomenon, the information has to be shared between the team members in a rapid and efficient manner. Thus, the last step of the ADVICE procedure regards dissemination of monitoring results. After the measurements sessions and the data processing steps, the results are automatically sent to a Web server. A dedicated webpage (“LiveData”, hosted at the address http://gmg.irpi.cnr.it/) has been set up in order present the results of the ADVICE procedure. At this page, accredited users may promptly consult the monitoring data few minutes after the end of the measurement sessions. For each monitored site, the web page is organized in different sections. The first page, called “Synthesis”, shows the last available update of the monitoring systems present in the area. In this page, selected plots and/or ©3DA representations are shown in order to provide rapidly to the end user about the current status. Secondly, users may explore other web-pages, each dedicated to the different instruments installed in the site with some representation that show the recorded history. In some case, the web platform is also used to share additional information, such as reports, Google™ Earth (kmz) files with details on the monitoring network, last monitoring bulletin, *etc.*

## 3. Operative Scenario: The Montaguto Earthflow, Southern Italy

**Figure 5.**
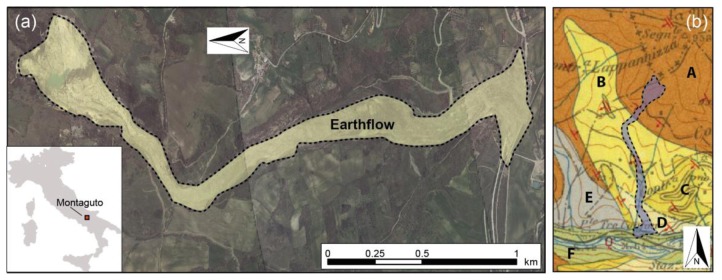
(**a**) Overview of Montaguto landslide; (**b**) Geological map of the Montaguto area. A: Flysch formation (Burdig. sup.—Langhiano Inf.); B: Argillaceous marl unit (Messin. Sup.—Plioc. Inf.); C: Arenitic unit (Mess. Inf.); D: Conglomeratic Unit; E: Ligurid unit; F: Alluvial deposits.

In the Montaguto area, southern Italy, *ca.* 100 km northeast from Naples, a large-scale earthflow was identified early in the 1950s (Figure 5). The instable area has a total length of about 3 km and the estimated total landslide volume is in the order of 4 millions of cubic meters [24,29].

In the spring of 2010, the landslide's toe reached the bottom of the valley with velocities ranging from 1 to 6 m/day. The mass wasting event of April 2010 severely damaged the SS90 road and covered approximately 300 m of an important railroad connecting Naples to Foggia. The railroad and road traffic was interrupted for about three months, causing problems for the residents and the local economy (details can be found in [30]). An integrated monitoring system was installed to assess the evolution of the landslide phenomenon, mainly to measure the surface movement of the earthflow and to help in the evaluation of the actions to be taken for assuring the safety of infrastructures involved. We provided support to the National Department of Civil Protection (DPC) for these activities mainly building an integrated monitoring network [24].

The Montaguto earthflow has been the operative scenario where the ADVICE procedures have been implemented for the first time, developed, thoroughly tested, and refined. In the following, we focus on the RTS network installed at the toe of the Montaguto earthflow, mainly for its important implications in terms of civil protection. At the moment of writing, we are operating this monitoring network and the associated EWS through the ADVICE procedures fully implemented as described in Section 2. However, this represents the point of arrival after a complex evolution lasted about three years, of which we will revisit the three main phases: (i) during the emergency context, to provide safety to the operators involved in the earthworks; (ii) after re-opening the road and the railway, to ensure safety for car and train circulation, as well as to provide details for the design of geotechnical remediation of the active landslide; (iii) after the mitigation activities.

### 3.1. Displacement Monitoring in the Emergency Scenario

**Figure 6.**
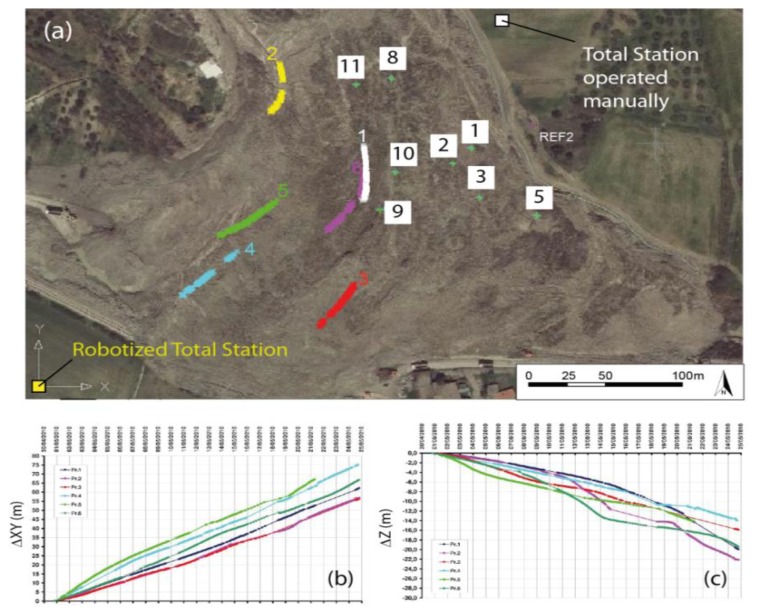
Representation of the displacement occurred at the landslide's toe during the emergency phase. (**a**) Monitoring network schema, with the traces drawn by the targets monitored via the RTS during the first month. Targets monitored via manual measurements (in the white boxes) show very little displacements; (**b**) and (**c**) are the time series of the planimetric and altimetric displacements, respectively, monitored via the RTS. These representations evidence a non-homogenous landslide evolution, which is very hard to understand by using only 2-D plots.

During the first emergency phases, the main goal was removing landslide material from the road and the railway. To this end, a multi-disciplinary emergency team was set up and coordinated by DPC, involving local authorities, earthworks operators, military, decision makers, and scientists [30]. The landslide's toe was highly unstable, as surface velocities were up to 6 m/day. Therefore, the safety conditions for workers involved in the removal operations were critical. In order to support DPC earthworks operations, as well as to control the response of the landslide toe to these activities, we set up a monitoring network based on Figure 6(a): (i) a RTS permanently installed on stable ground, and controlling the position of six optical targets located on the western part of the landslide's toe with revisit times of *ca.* 15 minutes; (ii) a second Total Station, operated manually every twelve hours, which controlled the position of eight further targets installed on the eastern side of the landslide's toe.

Estimated accuracies of the RTS measurements for this monitoring network configuration are in the order of ±1 mm. Monitoring results were initially presented to the partners in the standard form of time series graphs (Figure 6(b,c)). As stated in the introduction, the divulgation of monitoring results in this form to people with different technical/educational background present *in-situ* during the emergency phases may cause an inhomogeneous (and sometimes misleading) understanding of the current situation.

To improve the understanding of RTS results, we complemented time series plots with maps showing the position of monitored targets, as well as the indication of their traces on the surface (see Figure 6(a)). Moreover, as the displacements in the initial phase were very large, some of the benchmarks failed and were relocated and/or re-installed multiple times, and to ensure continuity to the time series we adopted an innovative approach based on the use of “virtual benchmarks” (details of this method can be found in [24]).

These were important adds-on mainly to communicate that the western portion of the landslide's toe was the most critical, with a peculiar kinematic behavior leading to secondary sliding effects in that area [24]. In any case, the graphic quality of the monitoring outputs was low, as they had to be produced quickly, directly in the field, and with very limited access to software and hardware resources. However, in the emergency phases we were also in the field, and could give clarifications about the monitoring results during daily briefings with the team involved in the emergency operations.

### 3.2. Monitoring after Road and Railway Re-Opening

At the end of July 2010, the first emergency earthworks were completed, and the road and the train circulation fully restored. Thus the DPC monitoring Presidium was progressively reduced. We maintained only the automatic RTS network, which was remotely controlled from our base office in Turin, northern Italy, more than 800 km away from the monitored area. The number and distribution of targets was updated to deal with the current condition of the landslide body, and with the main purpose of safeguarding cars and trains circulation. In addition, surface displacement data were used also by geologists and engineers involved in the geotechnical remediation, as a support to analyze the medium- and long-term evolution of the landslide, and to design proper protection and mitigation measures. Compared with the emergency phase, surface velocities at the landslide's toe were up to two orders of magnitude lower (*ca.* 0.05 m/day), with some accelerations associated with rain precipitations.

**Figure 7.**
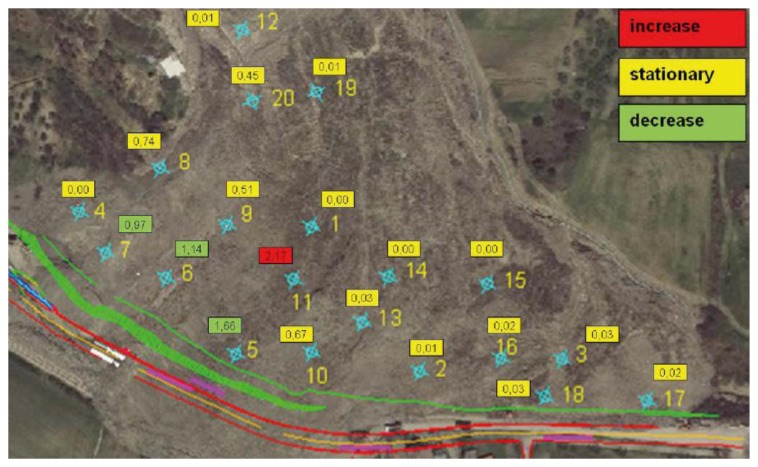
Example of the displacements synoptic map included in the bulletins. This representation can be considered as a precursor of the subsequent ©3DA representation.

The RTS monitoring results were shared daily with the team partners via bulletins, as well as extended reports sent on a monthly basis. As mentioned in Section 3.1, during the emergency context we noticed that conventional time series plots used for the divulgation of displacement monitoring were not adequate, considering the multi-disciplinary team involved in the operations. After the emergency, these problems were amplified by the fact that there was not a daily direct contact between the team partners. To deal with this issue, in the daily and monthly bulletins we complemented standard time series plots relevant to planimetric and altimetric displacements with a new form of map, where the displacements occurred in a specific period have been included in a numeric form close to the each monitored target (Figure 7). In addition, an self-explanatory color coding was adopted to represent areas of the landslide that were accelerating (red), stationary (yellow), or decelerating (green) in comparison with the previous reference period.

**Figure 8.**
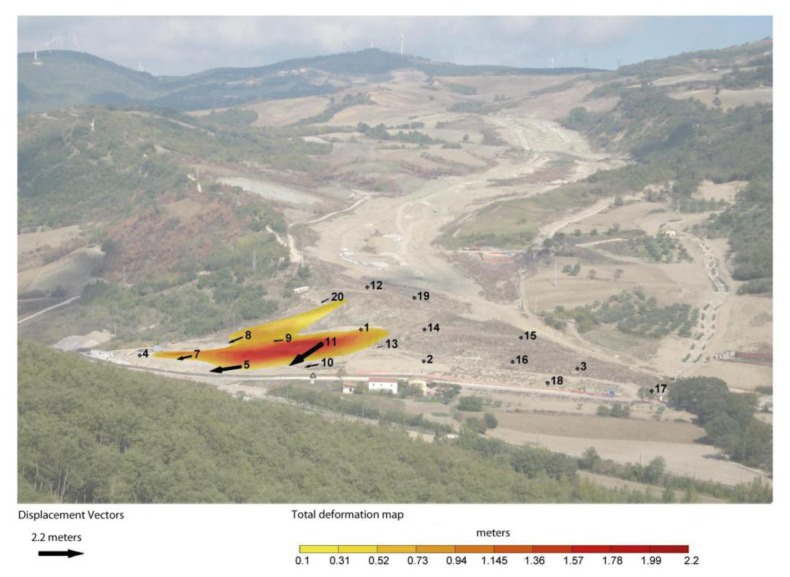
©3DA representation of the same dataset shown in Figure 7.

By using this representation it has been possible to generate a “synoptic” map of the RTS results, and communicate to the partners how the landslide's toe evolved over time in a rapid and efficient manner; however, the output was still generated “manually” for each bulletin and/or report. Following these considerations, we started developing a set of protocols and software procedures, which resulted in a straightforward method to produce automatically synoptic maps few minutes after each measurement session (*i.e.*, ©3DA, see also Section 2.3). Figure 8 shows the result obtained by applying the ©3DA algorithm to the displacements revealed in February 2011, which is the same dataset shown in Figure 7. This representation of the RTS monitoring data in near real time allowed for an immediate and clearer representation of the evolution of Montaguto earthflow phenomenon.

### 3.3. After Mitigation

**Figure 9.**
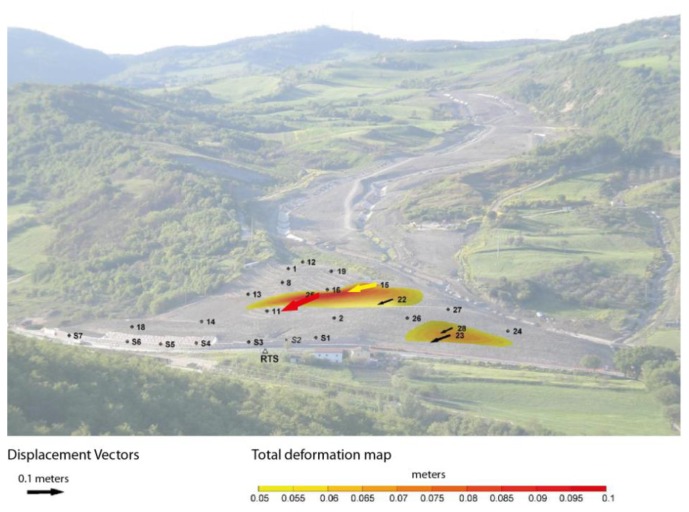
©3DA representation of ground displacements occurred in the period 10–17 March 2013 with indications of targets that overcome predefined warning (yellow) and alarm (red) surface velocity thresholds. This reactivation occurred after a rain precipitation of about 50 mm within one week. Note the targets installed on the earth reinforcing barriers, marked with “S”.

**Figure 10.**
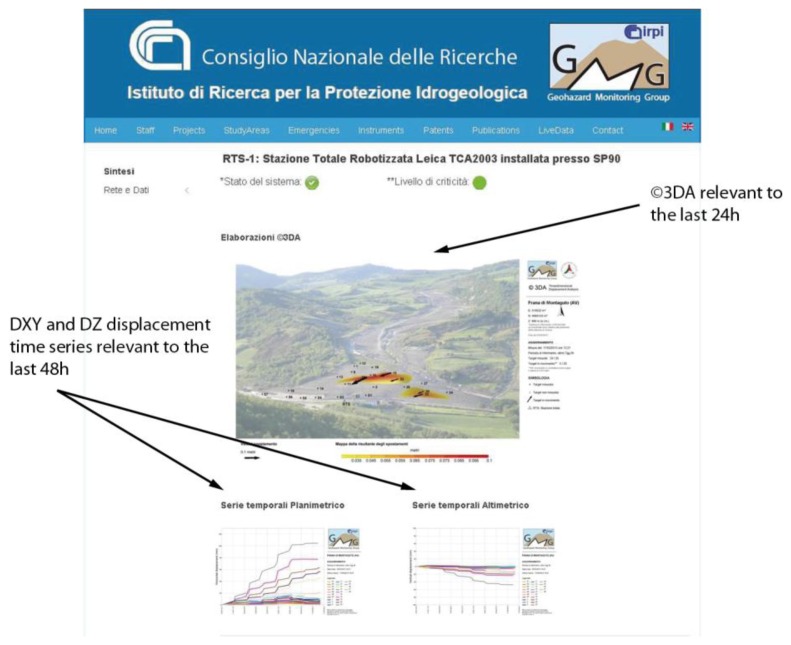
Example of the webpage dedicated to the near-real-time dissemination of monitoring data relevant to the Montaguto landslide.

Since May 2012 a new phase started, as the main remediation works on the most active portions of the landslide were completed. The landslide's toe has been stabilized mainly by reshaping the slope profile and consolidated through earth reinforcing barriers of the Erdox^®^ Terra type [31]. The RTS network has been further adapted to the new configuration of the slope, and monitoring targets were placed also on the consolidation barriers. Indeed, during this phase the RTS monitoring network has the key role to control the stability and effectiveness of the remedial measures. Moreover, potential reactivation of the landslide phenomena, for example during and/or after intense precipitation, has to be caught on time to avoid new hazards, and potentially further damages to the road and railway infrastructures. Ground deformation is now very limited, and thus the level of attention to displacement phenomena further reduced. At this stage, we implemented the possibility to display different colors for the vector arrows in the ©3DA outputs when predefined displacement and/or velocity thresholds (Figure 9). This allows to directly spot the areas potentially leading to a risk. In addition, when thresholds are overcome, ADVICE sends alert messages to the landslide experts managing the monitoring system via SMS and/or email. This procedure is very useful to control quiescent phenomena potentially affected by reactivation also in limited sectors, and thus to ensure safety conditions of the road and railway traffic. In order to facilitate the dissemination of monitoring results, we set up a dedicated webpage where people involved in the activities of this stage can have a direct access through the Internet (Figure 10).

## 4. Discussion

Early Warning Systems (EWS) are widely applied to manage and possibly reduce the hazard potential relevant to natural and/or anthropic phenomena. The large diffusion of EWS has been triggered by several factors, including the increasing availability of automatic and reliable monitoring sensors, as well as the coeval lowering of their cost. Following the UNISDR definition [32], EWS are: “The set of capacities needed to generate and disseminate timely and meaningful warning information to enable individuals, communities and organizations threatened by a hazard to prepare and to act appropriately and in sufficient time to reduce the possibility of harm or loss.” Therefore, monitoring sensors can be considered as the starting point of a multi-level workflow, which begins at the automatic acquisition of accurate measurements, continues through the translation of the data into a meaningful warning information, and ends with its dissemination. All the steps have to be performed “timely”, in order to allow for effective countermeasures. Thus, for that regarding EWS, the near-real-time and/or real-time monitoring concept cannot be restrained to the acquisition of the measurements, but should be appropriately extended to the entire workflow to ensure their efficiency.

In this work, we presented ADVICE as a new approach developed mainly to produce and disseminate in near-real-time a clear information derived from surface displacement data. ADVICE is a complex hardware and software infrastructure that aims to fill the gap between the level of development reached nowadays by displacement monitoring sensors and the tools used for the exploitation of the data obtained. The procedures implemented cover all the steps from the data acquisition, transfer, storage, validation, analysis, restitution and dissemination. Here we have shown how the ADVICE concept has been successfully applied to RTS, which are widely diffused monitoring instruments applied in monitoring activities relevant to different scenarios [23,25,33–35]; however, similar procedures could be also implemented in activities involving other sensors used to monitor displacements relevant to natural hazards and/or anthropic activities. In our experience, by using RTS the temporal interval of all the steps performed in ADVICE is negligible compared with the measurement revisit time and/or measurement session time. This allows disseminating “timely and meaningful warning information”, also in cases of landslide emergency scenarios characterized by rapid kinematic behavior [36].

One of the main strengths of our method is the capability to convert displacement measurements into a new typology of representation, *i.e.*, ©3DA, which allows a straightforward divulgation of the results also to people not used to deal with monitoring data. To some extent, the approach considered to develop ©3DA algorithm is very similar to the strategy used to present reliable rapid-response maps of earthquake ground motion, *i.e.*, the ShakeMaps® [37]. As for ShakeMaps® rapidly produced and disseminated after earthquakes at the USGS webpage, the goal of the ©3DA is to simplify and maximize the flow of information in landslide emergency scenarios given the diverse audience, which may include scientists, civil protection operators, authorities, media, population, *etc.*

We have shown how ADVICE has been developed and thoroughly tested during about three years in the Montaguto landslide, a large-scale earthflow that reached the bottom of the valley in 2010 and severely damaged the SS90 road, as well as a portion of the adjacent railway track. The evolution of ADVICE can be outlined by considering three different phases.

During the emergency, the RTS monitoring was issued to provide safety to the operators involved in the earthworks aimed at restoring the road and railway traffic. In that period, we already developed procedures to acquire and analyze the data in near-real-time, however, the restitution of the results was still poor, and the understanding restricted to experienced operators. Therefore, there was still a gap between the prompt availability of important information about the surface displacements of the landslide and the possibility to divulgate monitoring results to a broader audience. This problem was to some extent counterbalanced by our availability to give clarifications about the current situation directly in the field during the emergency operations. At the end of the first phase, the road and the railway were re-opened, the landslide was still moving at moderate rates, and RTS monitoring was expected to ensure safety for car and train circulation, *i.e.*, traffic had to be promptly interrupted in case of accelerations of the landslide. At this stage, the data end-users were not located in the field, but in different buildings, cities or even regions, thus there was not a direct interaction. The development and divulgation of the monitoring results through the ©3DA representation facilitated the interaction between partners, as the results could be presented clearly, without ambiguities, in a user-friendly manner, to ensure a correct understanding of the phenomenon. Moreover, in the same period we also developed an additional step to the ADVICE procedure, to perform short-term forecasting (STF) on the evolution of monitored targets by considering well-known methods used to infer the time of failure of a landslide [38–40]. These methods might be used to assist operators in defining the time span between the last measurement session and a critical state of the monitored target (potentially leading to failure), and provide a timely response in case of hazard increase. In most cases this information is very critical, thus the divulgation of STF results obtained with automatic procedures has been limited to internal use and/or to experienced users, which are aware of the limitations of these methods and can take into account not only displacement data, but also additional information from other parameters [41]. After the mitigation activities the RTS monitoring evolved again, and the EWS was issued to provide a prompt response in case of reactivation of the landslide phenomenon. Thus, ADVICE was set up to send alert messages via SMS and/or emails in case of the overcoming thresholds relevant to critical states. Moreover, the institutions involved in the territorial management and responsible for the safety of the road and railway needed a prompt response from the EWS, and they should be able to access monitoring data in near real-time. Hence, the fastest, and more efficient way to disseminate the results was to publish them in the Internet, and give access through a secure login procedure.

## 5. Conclusions

We presented ADVICE, an innovative procedure to manage and share near-real-time displacement monitoring data. At the moment, this procedure is applied in four different monitoring contexts, including: (i) the herein presented Montaguto earthflow, southern Italy; (ii) San Giovanni Profiamma, Foligno (PG), central Italy, where in April 2013 a landslide interrupted the important road that connects Rome to the Adriatic Sea through central Apennines (SS3 Flaminia); (iii) Mt. de la Saxe rock-slide, Courmayeur (AO), northern Italy, where a large deep-seated landslide menaces the infrastructures located at the bottom of the valley, including the local community; (iv) monitoring activities related to the recovering of the Costa Concordia vessel wreck, near the coast of the Il Giglio island, demonstrating that ADVICE has potential applications not only in landslide scenarios but also in different contexts, such as monitoring of buildings, dams, and/or other complex infrastructures. In all the above-mentioned operative scenarios we received positive responses from the different operators involved. Comments, suggestions and feedbacks received by operators with different needs and interests lead over the last years to an improvement and a fine tuning of the whole procedure. Moreover, we remark that the use of ADVICE in operative scenarios improved the communication between partners and operators involved in the monitoring activities and increased the efficiency of the EWS, consequently facilitating the decision-making chain also in critical situations. The ADVICE methodology in general, and more specifically its final step (website divulgation), it is useful to disseminate the information about the current situation of the landslide. However, we remind that false alarms due to inaccuracies in the data because of instrument malfunctions, physical changes at the measurement site, and/or very local/shallow reactivations may always occur. Thus, critical decisions in emergency landslide scenarios, as starting an evacuation and/or closing the traffic on a road/railway, cannot be based only on the results of topographic monitoring and carried out automatically. The added value of ADVICE is to provide a straightforward and common platform to share information between the involved operators/experts about the monitoring results in near-real-time. In emergency situations, alerts are automatically sent only to the experts, which have to revise and validate the monitoring results, and then carefully evaluate following actions in cooperation with authorities and decision makers.
